# SOCIAL DISCONNECTION CORRELATES OF A “WISH TO DIE” AMONG A LARGE COMMUNITY-DWELLING COHORT OF OLDER ADULTS

**DOI:** 10.1093/geroni/igae098.0289

**Published:** 2024-12-31

**Authors:** Mark Ward

**Affiliations:** Trinity College Dublin, Dublin, Dublin, Ireland

## Abstract

Social disconnection and deaths by suicide among older adults are both important public health concerns. In response to the “Epidemic of loneliness and isolation”, the Surgeon General’s Advisory has identified social connection as a cross-disciplinary research priority and developed a national strategy to advance greater social connection. A ‘Wish to Die’ (WTD) is predictive of future suicide attempt(s) and social disconnection has been identified as an important predictor of suicide-related thoughts and behavior. Data were from The Irish Longitudinal Study on Ageing (TILDA), a sister cohort of the Health and Retirement Study (HRS). TILDA is a large cohort of 8500 community-dwelling older adults aged 50+. Cross-sectional analyses of the association between numerous markers of social disconnection (loneliness, social isolation, living alone, marital status, social participation, volunteering, and attending religious service) and WTD were conducted. Four percent reported a WTD. Both functional and structural markers of social disconnection were associated with WTD. Among these, loneliness was the strongest risk factor while attendance of religious services was an important protective behavior. There is a strong association between social disconnection and a WTD among older adults. There is also a strong association between depression and a WTD, while attending religious services or similarly prosocial settings may protect older adults from experiencing negative thoughts about dying.

